# Addition of glomerular lesion severity improves the value of anemia status for the prediction of renal outcomes in Chinese patients with type 2 diabetes

**DOI:** 10.1080/0886022X.2021.2009862

**Published:** 2022-02-21

**Authors:** Lijun Zhao, Qianqian Han, Li Zhou, Lin Bai, Yiting Wang, Yucheng Wu, Honghong Ren, Yutong Zou, Shuangqing Li, Qiaoli Su, Huan Xu, Lin Li, Zhonglin Chai, Mark E. Cooper, Nanwei Tong, Jie Zhang, Fang Liu

**Affiliations:** a Department of Nephrology, Laboratory of Diabetic Kidney Disease, Centre of Diabetes and Metabolism Research, West China Hospital of Sichuan University, Chengdu, Sichuan, China; b Department of General Practice, West China Hospital of Sichuan University, Chengdu, Sichuan, China; c Department of Pathology, West China Hospital of Sichuan University, Chengdu, Sichuan, China; d Histology and Imaging Platform, Core Facility of West China Hospital, Sichuan University, Chengdu, Sichuan, China; e Department of Diabetes, Central Clinical School, Monash University, Melbourne, Australia; f Division of Endocrinology, West China Hospital of Sichuan University, Chengdu, Sichuan, China; g Key Laboratory of Transplant Engineering and Immunology, NHFPC; Regenerative Medicine Research Center, West China Hospital of Sichuan University, Chengdu, Sichuan, China

**Keywords:** Diabetic nephropathy, anemia, glomerular classification, end-stage renal disease

## Abstract

We aimed to determine the utility of biopsy data and anemia for the prediction of renal outcomes in Chinese patients with type 2 diabetes. In total, 441 Chinese patients with type 2 diabetes and biopsy-confirmed diabetic nephropathy (DN) were enrolled in a retrospective study. Their renal pathology was assessed using the Renal Pathology Society system. Cox proportional hazards models were used to estimate hazard ratios (HRs) for end-stage renal disease (ESRD), and immunofluorescence staining was used to assess the expression of hypoxia-inducible factor (HIF)-α in patients’ kidneys. We found that glomerular pathology classification was an independent pathological predictor of low hemoglobin concentration, according to linear and logistic regression analyses. Each 1 g/dL decrease in baseline hemoglobin concentration was associated with a 42% higher risk of an adverse renal outcome, after adjustment for clinical and pathologic covariates. In patients with severe glomerular lesions, the risk of progression to ESRD was significantly higher if mild or moderate/severe anemia was present, but in patients with mild glomerular lesions, the risk was only significantly higher in those with moderate or severe anemia than in the absence of anemia. Harrell’s C Concordance was improved, but the Akaike information criterion was worsened by adding the glomerular pathology classification to the use of anemia status and clinical data. Immunofluorescence staining revealed that renal HIF-1α and HIF-2α expression was significantly higher in classes II–IV than class I. Thus, the addition of glomerular pathology classification increases the value of anemia status for the prediction of the progression to ESRD.

## Introduction

The prevalence of diabetes mellitus has increased rapidly in recent years, such that an estimated 415 million people worldwide lived with diabetes mellitus in 2015 and, by 2040, 642 million people are expected to have diabetes, of whom 30%–40% will develop diabetic nephropathy (DN). DN has become the major cause of chronic kidney disease (CKD) in the United States and many other developed and developing countries, such that it now accounts for approximately half of the end-stage renal disease (ESRD) burden worldwide.

Anemia is a frequent complication of DN and it is often more severe and develops earlier in patients with DN than in patients with CKD of other etiologies [1,2]. A previous cross-sectional study showed that the prevalence of anemia increases with the stage of CKD, from 8.4%–22.4% in stage 1 CKD to 50.3%–79.2% in patients with stage 4 CKD [2,3]. The severity of anemia closely correlates with both the progression of CKD and patient survival [4]. Furthermore, the severity of anemia correlates with the prevalence of cardiovascular and all-cause mortality in patients with type 2 diabetes, independent of the presence of CKD [5]. However, these relationships were established without considering the effect of hemoglobin concentration (Hb) and renal pathology on renal outcomes.

Hypoxia is a classic stimulus for erythropoiesis. Hypoxia-inducible factors (HIFs) are key mediators of a broad spectrum of cellular responses to hypoxia and are essential for erythropoiesis under normal and hypoxic stress conditions. A lack of HIF in mice results in anemia [6,7], and *EPAS1* haplotypes, which affect the production of HIF-2α, are associated with low Hb in Tibetans [8,9]. However, the role of HIFs in DN is disputed. The renin-angiotensin system (RAS) and the endothelin system are overactive in DN, which results in high expression of angiotensin-II and endothelin-1, causing a prolonged period of vasoconstriction, and thus tissue ischemia and hypoxia. Studies have also shown that HIF-1α expression is high in patients with diabetes and kidney injury [10] and that angiotensin-II increases the expression of HIF-1α in renal tubular epithelial cells *in vitro* [11]. Therefore, it is necessary to characterize the expression of HIFs during the various stages of CKD and its association with anemia.

In the present study, we aimed to determine the effects of indices of renal pathology and anemia on the renal outcomes of Chinese patients with type 2 diabetes, while correcting for their baseline Hb. We also used immunofluorescence staining to characterize the expression of HIFs in patients with DN.

## Materials and methods

### Patient selection and study design

**Figure 1.**
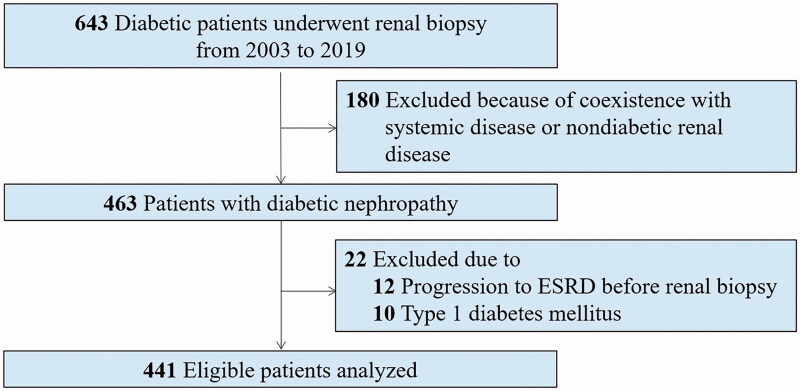
Flowcharts of participants in this study.

Among the 643 patients with diabetes who underwent renal biopsy at our hospital from January 2004 to April 2019, 441 patients with type 2 diabetes and biopsy-confirmed DN were considered eligible and were enrolled in the longitudinal observational study. The indications for renal biopsy were diabetes mellitus with renal damage, presence of obvious glomerular hematuria, short duration of diabetes, and sudden-onset overt proteinuria [9]. Diabetes mellitus was diagnosed based on American Diabetes Association criteria [12]. DN was defined in accordance with the criteria described in An et al. [13], and was diagnosed by at least two renal pathologists and/or nephrologists using Tervaert’s classification system [14]. Exclusion criteria were the presence of coexisting nondiabetic kidney diseases such as IgA nephropathy, systemic conditions including antineutrophil cytoplasmic antibodies (which are associated with vasculitis), anti-glomerular basement membrane disease, lupus nephritis, type 1 diabetes, and progression to ESRD prior to renal biopsy (Figure 1). All patients provided written informed consent, and the study was approved by the institutional review board of the West China Hospital of Sichuan University.

### Clinical and laboratory data

Baseline demographic and clinical data, including age, sex, body mass index (BMI), smoking status, hemoglobin A1c (HbA1c), presence of diabetic retinopathy, 24-h proteinuria, and use of renin-angiotensin-aldosterone system blockade were collected from the electronic medical records at the time of renal biopsy. The estimated glomerular filtration rate (eGFR) was evaluated using the Chronic Kidney Disease Epidemiology Collaboration formula [15,16]. The Hb concentration was measured by the resistance method using System XE-2100. Serum ferritin was measured with an immunoradiometric assay. Serum iron and total iron-binding capacity (TIBC) were measured with a modified automated AAII-25 colorimetric method. Transferrin saturation (TSAT) was calculated as (iron/TIBC) × 100% [16]. During the follow-up period, patients attended follow-up appointments 2–4 times annually, depending on their clinical condition.

### Definition of anemia

At the population level and in clinical practice, Hb concentration is the most common hematological assessment method used and the most common indicator used to define anemia [17]. The degree of anemia was defined according to the World Health Organization (WHO) criteria [18]: non-anemia, man (15 years of age and above) ≥13.0 g/dL, nonpregnant women (15 years of age and above) ≥12.0 g/dL; mild anemia, man 11.0–12.9 g/dL, nonpregnant women 11.0–11.9 g/dL; moderate anemia, man or nonpregnant women 8.0–10.9 g/dL; severe anemia, man or nonpregnant women <8.0 g/dL.

### Renal pathology

Renal biopsy samples were prepared for light microscopy, immunofluorescence, and electron microscopy using West China Hospital’s standard procedures. For light microscopy examination, renal specimens were stained with hematoxylin and eosin, periodic acid-Schiff, Masson’s trichrome, and periodic acid-Schiff silver methenamine. The original immunofluorescence microscopy and electron microscopy results were used to confirm the diagnosis of DN. All light microscopy pathological findings were defined and classified in accordance with the Renal Pathology Society (RPS) DN classification system [14]. The histological scoring under light microscopy or electron microscopy were evaluated by two neuropathologists, who were blinded to the clinical data and renal outcomes.

### Renal outcomes

The renal outcome was defined as the progression to ESRD. The ESRD was indicated by an eGFR <15 mL/min/1.73 m^2^, or the commencement of renal replacement therapy [19,20]. All patients were followed until April 2020.

### Immunofluorescence staining

Sections (3-μm thickness) cut from 10% formalin-fixed, paraffin-embedded kidney samples from patients with different RPS glomerular classes were used for immunostaining for HIF-1α or HIF-2α. The process was described detailed previously [21]. Briefly, heat-induced antigen retrieval (pH 9.0) was performed in preparation for incubation with anti-HIF-1α mouse monoclonal antibody (NB100-131, Novus Biologicals, USA) or anti- HIF-2α rabbit monoclonal antibody (NB100-122, Novus Biologicals, USA). After washing with PBS, the sections were incubated with secondary antibodies (Alexa Fluor 647 or Dy Light conjugated 550) for 60 min at 37 °C and then stained with DAPI (Calbiochem) according to the manufacturer’s instructions. Confocal laser scanning microscope and FV10-ASW software were used to analyze the tissue sections. HIF-1α or HIF-2α staining quantification was determined as a ratio of staining positive area/cortex area.

### Statistical analysis

Continuous variables are expressed as means ± standard deviations (SDs), if normally distributed, or as medians and interquartile ranges (IQRs), if not. Categorical variables are expressed as counts and percentages. Differences between continuous variables were analyzed using one-way ANOVA, followed by the Bonferroni or Tukey methods for multiple comparisons, or the Kruskal-Wallis *H*-test, as appropriate [19]. Categorical variables were analyzed using the chi-square test or Fisher’s exact test.

Univariate and multivariate linear regression analyses and logistic regression analyses were used to assess the cross-sectional relationships between baseline Hb and indices of kidney pathology. Survival curves were generated using the Kaplan-Meier method and the log-rank test was performed. Univariate and multivariate Cox proportional hazards models were used to estimate the hazard ratios (HRs) associated with Hb for the prediction of renal outcomes [22]. Data for 24-h proteinuria were missing for 10 individuals. Therefore, we assessed the differences in clinical parameters between patients with or without missing values to check whether the missing data were randomly distributed. We then used multiple imputation methods for the multivariable models. The proportional hazard assumption in the Cox model was tested to check whether the dataset satisfied the basic assumptions of Cox analyses. Then, three Cox proportional hazards models were used to calculate HRs and 95% confidence intervals (CIs) for renal outcomes. In the three models, each HR was adjusted for the age and sex of the patient at the time of renal biopsy. The first multivariable model was adjusted for age and sex alone, the second for age, sex, and clinical covariates (eGFR, 24-h proteinuria, serum albumin, and HbA1c at the time of renal biopsy) as continuous variables, and the third for the aforementioned factors plus renal pathologic covariates that had *p* values of <0.05 in the univariate models. Age and sex were chosen based on biological plausibility and the clinical covariates were chosen as potential confounders because they were statistically significant in univariate models or have previously been shown to be associated with a higher risk of adverse renal outcomes [23,24]. Parameters with *p* < 0.05 in the third model were considered to be potentially useful prognostic indicators.

The incremental prognostic value of including RPS glomerular classification and serum Hb in the model, compared with a model that only contained renal functional parameters and pathologic findings, was analyzed by calculating Harrell’s C-statistic and the likelihood ratio, and assessing the Akaike information criterion (AIC) [25]. Statistical analyses were performed using Stata version 14.0 (StataCorp LLC, College Station, TX, USA) or SAS version 9.4 (SAS Institute Inc., Cary, NC, USA). *p* < 0.05 was deemed to indicate statistical significance.

## Results

### Baseline clinical and pathologic characteristics of the patients, according to their Hb

Of the 441 patients enrolled in the study, 70.5% were male (*n* = 311). At the time of the biopsy, their mean Hb was 11.9 g/dL. The prevalence of anemia was 31.2% for those in CKD stage 1, 64.2% for those in CKD stage 2, 81.2% for those in CKD stage 3, and 86.2% for those in CKD stage 4. Thus, the prevalence of anemia increased with advances in the CKD stage. The median baseline eGFR was 59.1 mL/min/1.73 m^2^ (IQR 42.0–90.4 mL/min/1.73 m^2^) and the median baseline 24-h proteinuria was 4.2 g/day (IQR 2.0–7.5 g/day). Using the World Health Organization’s definition of anemia, 154 (34.9%) patients were not anemic, 121 (27.4%) had mild anemia, 160 (36.3%) had moderate anemia, and 6 (1.4%) had severe anemia. Because few of the patients had severe anemia, those with moderate or severe anemia were subsequently analyzed as a single group. The demographics and baseline characteristics of the patients, categorized according to their Hb, are shown in Table 1. Compared with those without anemia, the patients with anemia had higher systolic blood pressure and more severe proteinuria, but lower baseline body mass index, eGFR, HbA1c, and fasting plasma glucose and serum albumin concentrations. The serum ferritin, iron, TIBC, TSAT concentrations were decreased when the serum Hb concentration decreased.

**Table 1.** Baseline clinical pathologic features of patients according to the baseline hemoglobin concentration.

Characteristics	All (*n* = 441)	No anemia (*n* = 154)	Mild anemia (*n* = 121)	Moderate/Severe anemia (*n* = 166)	*p*
Age, mean (SD), y	51 (9)	51 (10)	52 (9)	51 (9)	0.48
Sex, male, *n* (%)	311 (70.5)	124 (80.5)	93 (76.9)	94 (56.6)	<0.01
Smoking, Never/Ex/Current, (*n*)	238/67/136	77/21/56	58/18/45	103/28/35	0.02
BMI, mean (SD), kg/m^2^	25.2 (3.6)	25.6 (3.8)	25.3 (3.3)	24.6 (3.7)	0.03
SBP, mean (SD), mmHg	146 (23)	142 (23)	147 (22)	148 (25)	0.04
DBP, mean (SD), mmHg	86 (13)	88 (12)	84 (12)	86 (14)	0.10
History of DR, *n* (%)	227 (51.5)	58 (37.7)	68 (56.2)	101 (60.8)	<0.001
Duration of diabetes, median (IQR), months	96 (36–132)	84 (36–120)	120 (60–156)	96 (36–132)	<0.01
HbA1c, median (IQR), %	7.29 (6.3–8.4)	7.74 (6.7–9.2)	7.2 (6.4–8)	6.8 (6–7.8)	<0.001
FPG, median (IQR), mg/dL	130.1 (101.2–174.4)	139.4 (104.9–176.4)	128.5 (109.6–171.9)	123.9 (91.3–173.7)	0.03
Serum Hb, mean (SD), g/dL	11.9 (2.7)	14.8 (1.9)	11.8 (0.8)	9.36 (1.1)	<0.001
Serum albumin, mean (SD), g/L	33.9 (7.8)	38 (7.4)	33.5 (7.3)	30.5 (6.7)	<0.001
eGFR, median (IQR), mL/min/1.73 m^2^	59.1 (42–90.4)	89 (57.8–109.5)	57 (42.9–82.7)	48.1 (32.2–63.2)	<0.001
24-h proteinuria, median (IQR), g/d	4.2 (2–7.5)	2.81 (1.05–4.82)	4.3 (2.24–8.04)	5.71 (3.15–8.85)	<0.001
Ferritin, median (IQR), ng/mL	311.0 (158.2–575.0)	609.5 (195.3–1060.5)	375.2 (131.8–509.6)	264.2 (154–522.3)	0.16
Fe, median (IQR), mmol/L	10.1 (8.1–15.2)	20.2 (17–23.8)	12.3 (8.4–16.3)	9.4 (7.3–12.6)	<0.001
TIBC, median (IQR), mmol/L	35.4 (29.7–42.2)	41.6 (37.1–51.8)	39.2 (29.3–45.4)	33 (29.1–40)	0.01
TSAT, median (IQR), %	30.88 (22.54–41.33)	43.2 (34.7–78.2)	34.9 (24.6–44.3)	28.9 (20.5–34.6)	<0.01
RAAS inhibitor, *n* (%)	347 (78.7)	135 (87.7)	96 (79.3)	116 (69.9)	<0.01
Statins, *n* (%)	273 (61.9)	98 (63.6)	80 (66.1)	95 (57.2)	0.27
ESA/ iron supplementation, *n* (%)	56 (12.8)	0 (0)	8 (6.6)	48 (28.9)	<0.001
RPS classification^†^, *n* (%)					<0.001
I	18 (4.1)	16 (10.4)	2 (1.7)	0 (0)	
Iia	83 (18.8)	66 (42.9)	9 (7.4)	8 (4.8)	
Iib	69 (15.6)	26 (16.9)	30 (24.8)	13 (7.8)	
III	200 (45.4)	31 (20.1)	62 (51.2)	107 (64.5)	
IV	71 (16.1)	15 (9.7)	18 (14.9)	38 (22.9)	
IFTA^†^, *n* (%)					<0.001
Score 0	16 (3.6)	12 (7.8)	2 (1.7)	2 (1.2)	
Score 1	217 (49.2)	95 (61.7)	60 (49.6)	62 (37.3)	
Score 2	158 (35.8)	42 (27.3)	47 (38.8)	69 (41.6)	
Score 3	50 (11.3)	5 (3.2)	12 (9.9)	33 (19.9)	
Interstitial inflammation^†^, *n* (%)					<0.001
Score 0	14 (3.2)	12 (7.8)	2 (1.7)	0 (0)	
Score 1	317 (71.9)	125 (81.2)	82 (67.8)	110 (66.3)	
Score 2	110 (24.9)	17 (11.0)	37 (30.6)	56 (33.7)	
Arteriosclerosis^†^, *n* (%)					<0.01
Score 0	58 (13.2)	33 (21.4)	13 (10.7)	12 (7.2)	
Score 1	215 (48.8)	72 (46.8)	59 (48.8)	84 (50.6)	
Score 2	168 (38.0)	49 (31.8)	49 (40.5)	70 (42.2)	
Arteriolar hyalinosis^†^, *n* (%)					<0.01
Score 0	57 (12.9)	33 (21.4)	11 (9.1)	13 (7.8)	
Score 1	126 (28.6)	43 (27.9)	37 (30.6)	46 (27.7)	
Score 2	258 (58.5)	78 (50.6)	73 (60.3)	107 (64.5)	

Data are presented as the mean (standard) for continuous variables with symmetric distribution, median (25th–75th percentiles) for continuous variables with asymmetric distribution, or percentages for categorical variables. ^†^Defined by RPS Diabetic Nephropathy Classification.

SD, standard deviation; IQR, interquartile range; BMI, body mass index; SBP, systolic blood pressure; DBP, diastolic blood pressure; DR, diabetic retinopathy; HbA1c, hemoglobin A1c; FPG, fasting plasma glucose; eGFR, estimated glomerular filtration rate; RAAS, renin-angiotensin-aldosterone system; ESA, erythropoiesis-stimulating agent; TIBC, total iron-binding capacity; TSAT, transferrin saturation; RPS, Renal Pathology Society; IFTA, interstitial fibrosis and tubular atrophy.

Of note, only 8 (1.8%) patients have a presence of fewer than ten glomeruli per biopsy specimen. Their clinical characteristics have no significant difference between patients with more than ten glomeruli per slice and those who have less than ten glomeruli per slice (Supplementary Table 1). According to the RPS DN classification, there were 18 (4.1%) patients in class I, 83 (18.8%) patients in class IIa, 69 (15.6%) patients in class IIb, 200 (45.4%) patients in class III, and 71 (16.1%) patients in class IV. Interstitial fibrosis and tubular atrophy (IFTA) scores of 0, 1, 2, and 3 were obtained for 16 (3.6%), 217 (49.2%), 158 (35.8%), and 50 (11.3%) patients, respectively. Compared with patients without anemia, those with mild, moderate, or severe anemia had more severe renal pathologic lesions, including RPS glomerular lesions, IFTA, interstitial inflammation, arteriosclerosis, and arteriolar hyalinosis (Table 1).

### Correlations between Hb and pathological findings

To investigate the relationships between Hb and pathological findings, univariate linear regression analysis was performed, which showed that RPS DN glomerular lesions, IFTA lesions, interstitial inflammation, arteriosclerosis, and arteriolar hyalinosis were significantly associated with a low Hb. However, in multivariate linear regression analysis, only RPS glomerular lesions remained significantly associated with a low Hb (Supplementary Table 2).

To further clarify the relationships between pathologic indices and anemia, univariate and multivariate binary logistic regression analysis was used (Supplementary Table 2). Univariate logistic regression analysis showed that RPS glomerular lesions, IFTA, interstitial inflammation, arteriosclerosis, and arteriolar hyalinosis were significantly associated with moderate or severe anemia. However, in the multivariate binary logistic regression analysis, only RPS glomerular lesions (odds ratio [OR] 2.18, 95% CI 1.75–2.73) were significantly associated with moderate or severe anemia, after adjustment for age, sex, baseline eGFR, and proteinuria.

### Baseline clinical characteristics of the patients, categorized according to Hb and the severity of their glomerular lesions

The severity of the glomerular lesions closely correlated with baseline Hb, and therefore the clinical and pathologic characteristics of the patients were compared after stratification according to their baseline Hb and RPS glomerular classification. The RPS glomerular classification was further categorized as mild changes (classes I and II) and severe changes (classes III and IV). The proportions of patients with severe glomerular lesions were 29.9% (46 of 154) in the no anemia group, 66.1% (80 of 122) in the mild anemia group, and 87.3% (145 of 166) in the moderate or severe anemia group. The baseline clinical characteristics of the patients, categorized according to their Hb and the severity of their glomerular lesions, are displayed in Supplementary Table 3. This analysis showed that low serum albumin concentration, low HbA1c, low eGFR, and more frequent use of erythropoiesis-stimulating agent (ESA) or iron supplementation were associated with low Hb when either mild or severe glomerular lesions were present (Supplementary Table 3).

### Risk factors for renal outcomes

**Figure 2.**
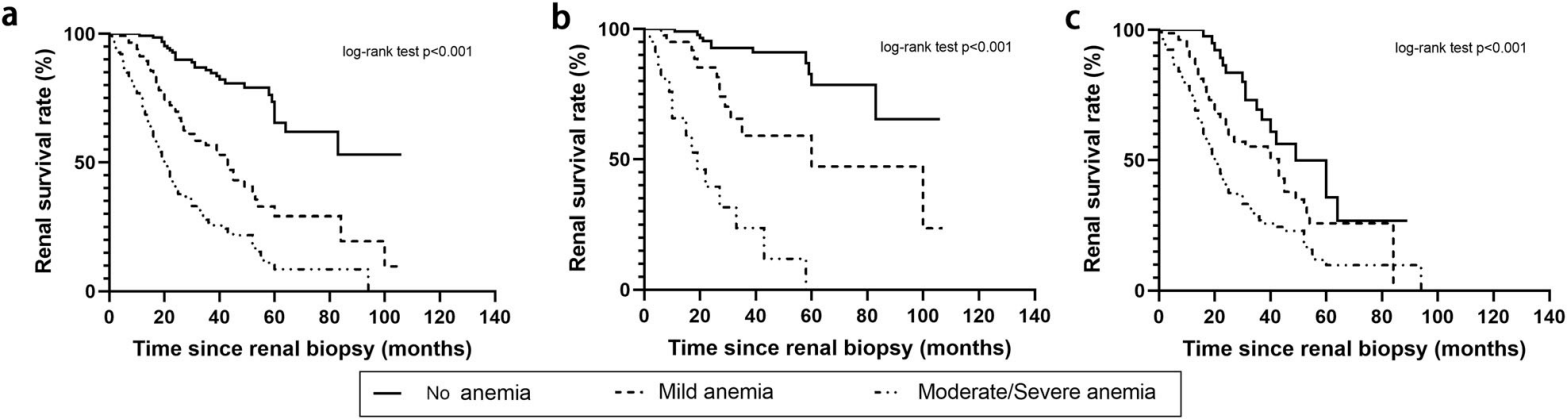
Kaplan-Meier survival curves for renal outcome according to baseline hemoglobin concentration by Renal Pathology Society glomerular lesions. (a) Kaplan-Meier survival curves for renal outcome stratified by the severity of anemia in the total 441 patients. (b) Kaplan-Meier survival curves for renal outcome stratified by the severity of anemia in 170 patients with mild glomerular lesions. (c) Kaplan-Meier survival curves for renal outcome stratified by the severity of anemia in 271 patients with severe glomerular lesions.

Of the 441 participants, 200 (45.4%) progressed to ESRD during a median follow-up period of 28 months. In the no anemia, mild anemia, and moderate or severe anemia groups, 28 (14.0%), 54 (27.0%), and 118 (59.0%) patients progressed to adverse renal outcomes, respectively. Kaplan-Meier survival analysis demonstrated that the 5-year survival rates of patients were 65.2% in the no anemia group, 30.2% in the mild anemia group, and 10.5% in the moderate or severe anemia group (*p* < 0.001) (Figure 2a). Univariate Cox proportional hazard analysis showed that mild anemia and moderate or severe anemia were significantly associated with the progression to adverse renal outcomes (Table 2). After adjustment for clinical and renal pathologic covariates, both mild anemia (adjusted HR 3.58, 95% CI 2.24–5.72) and moderate or severe anemia (adjusted HR 8.16, 95% CI 5.31–12.55) were significantly associated with adverse renal outcomes, compared with the no anemia group. Specifically, each 1 g/dL decrease in baseline Hb was associated with a 42% (95% CI 1.33–1.51) higher risk of an adverse renal outcome.

**Table 2.** Univariable and multivariable Cox proportional hazard models by the severity of anemia and glomerular lesions at the renal endpoint.

	All				Mild glomerular lesions (classes I + II)			Severe glomerular lesions (classes III + IV)		
Variables	No anemia	Mild anemia	Moderate/ Severe anemia	Hb (−10g/L)	No anemia	Mild anemia	Moderate/ Severe anemia	No anemia	Mild anemia	Moderate/ Severe anemia
Univariate Models HR (95% CI)	1 (reference)	3.33 (2.1–5.26)	7.69 (5.07–11.68)	1.39 (1.31–1.49)	1 (reference)	3.82 (1.69–8.63)	6.47 (8.17–13.77)	1 (reference)	1.79 (1.01–3.15)	3.29 (1.96–5.51)
*p* value		<0.001	<0.001	<0.001		<0.001	<0.001		0.04	<0.001
Multivariable model 1^a^ HR (95% CI)	1 (reference)	1.86 (1.15–3.03)	3.33 (2.08–5.31)	1.20 (1.11–1.29)	1 (reference)	2.02 (0.78–5.2)	4.36 (3.03–11.1)	1 (reference)	1.94 (1.07–3.52)	3.38 (1.91–6.00)
*p* value		0.01	<0.001	<0.001		0.15	<0.001		0.03	<0.001
Multivariable model 2^b^ HR (95% CI)	1 (reference)	3.58 (2.24–5.72)	8.16 (5.31–12.55)	1.42 (1.33–1.51)	1 (reference)	1.60 (0.59–4.37)	2.48 (2.52–9.2)	1 (reference)	2.04 (1.10–3.76)	3.52 (1.96–6.32)
*p* value		<0.001	<0.001	<0.001		0.36	<0.001		0.02	<0.001

^a^Adjusted for age, sex. ^b^Adjusted for the parameters in a multivariable model a plus baseline eGFR, 24-h proteinuria, HbA1c, serum albumin concentration, diabetic retinopathy and renal pathological parameters (Renal Pathology Society glomerular classifications, interstitial fibrosis and tubular atrophy, interstitial inflammation, arteriosclerosis and arteriolar hyalinosis).

HR, hazard ratio; CI, confidence interval; eGFR, estimated glomerular filtration rate; HbA1c, hemoglobin A1c.

Figures 2b,c shows the survival curves for the progression to adverse renal outcomes, according to the baseline Hb and the severity of glomerular lesions. For patients with mild glomerular lesions, the 5-year survival rate of patients was 80.1% in the no anemia group, 45.2% in the mild anemia group, and 0% in the moderate or severe anemia group, respectively (*p* < 0.001, Figure 2b). In patients with severe glomerular lesions, the 5-year survival rate of patients was 35.7% in then no anemia group, 25.0% in the mild anemia group, and 10% in the moderate or severe anemia group, respectively (*p* < 0.001, Figure 2c).

Table 2 shows the adjusted HRs for adverse renal outcomes, categorized according to their baseline Hb and the severity of their glomerular lesions, after adjustment for clinical and renal pathologic covariates. The patients without anemia but with mild or severe glomerular lesions served as the reference group. The risks of progression to ESRD were significantly higher in patients with mild anemia (adjusted HR 2.04, 95% CI 1.10–3.76; *p* = 0.02) or with moderate or severe anemia (adjusted HR 3.52, 95% CI 1.96–6.32; *p* < 0.001), and severe glomerular lesions (classes III and IV). However, the risk was significantly higher only in the moderate or severe anemia group (adjusted HR 2.48, 95% CI 2.52–9.20; *p* < 0.001) when the patients had mild glomerular lesions (classes I, IIa, or IIb).

Supplementary Figure 1 demonstrates the combined effect of baseline Hb and glomerular lesions on the risk of progression to ESRD, calculated using multivariate Cox proportional hazard analysis. The patients without anemia but with mild glomerular lesions served as the reference group. In the full cohort, the risk of progression to ESRD was significantly higher in patients with anemia and severe glomerular lesions than in those without anemia and with mild glomerular lesions. Among the patients with mild glomerular lesions, the adjusted HRs for adverse renal outcomes increased from 3.51 in those with mild anemia to 4.73 in those with moderate or severe anemia. Among those with severe glomerular lesions, the adjusted HRs for adverse renal outcomes increased from the no anemia group (HR 3.46) to the mild anemia group (HR 6.47) and the moderate or severe anemia group (HR 10.72), compared with the patients without anemia and with mild glomerular lesions.

### Incremental prognostic value of RPS glomerular lesion severity for the prediction of adverse renal outcomes

The incremental prognostic value of low Hb in addition to baseline clinical covariates to predict adverse renal outcomes was assessed using global chi-square analysis, the results of which are presented in Table 3. The Harrell’s C Concordance statistic for a model that only contained clinical variables (age, sex, baseline eGFR, and proteinuria) was improved by the addition of anemia, which implies that the adjusted model was superior (Harrell’s C-index was 0.7535 and 0.7836, respectively). Furthermore, Harrell’s C-index increased to 0.7958 when the severity of the glomerular lesions was added to the one that included anemia. Additionally, the AIC was reduced by the addition of the IFTA score to the basic model. The AIC of adding IFTA to the basic model was slightly higher than that of adding RPS classification. The model containing the clinical variables plus the RPS classification and anemia yielded the lowest AIC of the four models (Table 3), which implies that the addition of the severity of the glomerular lesions improves the prognostic power of a model that comprises clinical parameters and the severity of anemia.

**Table 3.** The incremental prognostic value of glomerular lesions on anemia for predicting the risk of progression to ESRD.

Statistics	Model 1^a^	Model 1 + anemia^b^	Model 1 + RPS^c^	Model 1 + IFTA^d^	Model 1 + anemia + RPS^e^
Harrell's C Concordance statistics	0.7535	0.7836*	0.7742*	0.7612*	0.7958*
LR *χ*^2^	138.91	185.15*	180.83	169.58*	189.85*
AIC	1998.252	1956.013	1960.851	1972.430	1953.316

^a^Model 1 included age, sex, baseline estimated glomerular filtration rate, and 24-h proteinuria. ^b^Model included the covariates in model 1 plus the severity of anemia. ^c^Model included the covariates in model 1 plus the RPS glomerular classifications. ^d^Model included the covariates in model 1 plus IFTA score.^ e^Model included the covariates in model 1 plus the severity of anemia and RPS glomerular classification.

LR *χ*^2^, likelihood ratio Chi-square statistics; AIC, Akaike information criterion. RPS, renal pathology society; IFTA, interstitial fibrosis and tubular atrophy. *p < 0.05 (versus Model 1).

### Expression of HIF-1α and HIF-2α in kidney biopsy samples

**Figure 3.**
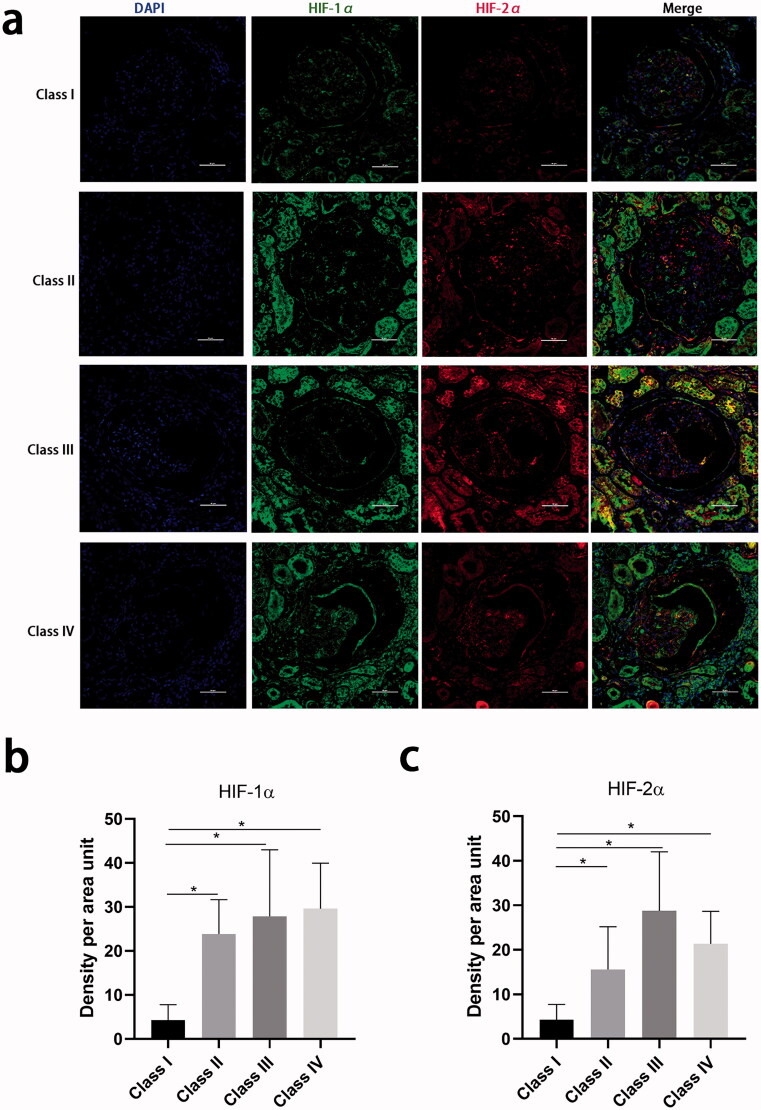
Hypoxia-inducible factor-1α and hypoxia-inducible factor-2α staining in kidney biopsy specimens. (a) Tissue hypoxia-inducible factor-1α by immunofluorescence in kidney tissue from different glomerular classifications defined by Renal Pathology Society. 400×, Bar = 50 µm. (b) Quantification of the hypoxia-inducible factor-1α staining in the whole tissue. (c) Quantification of the hypoxia-inducible factor-2α staining in the whole tissue (∗*p* < 0.05).

To assess HIF-1α and HIF-2α expression in the kidney, immunofluorescence staining for each protein was performed on kidney biopsy samples from 21 patients with diabetes and RPS glomerular classes I, II, III, or IV. The baseline clinical characteristics of the patients are shown in Supplementary Table 4. Their median eGFR was 58 mL/min/1.73 m^2^ and their median baseline 24-h proteinuria was 3.6 g/d, which are comparable to the values for the entire cohort. The serum Hb decreased slightly from class I to class IV. Figure 3 shows that more HIF-1α and HIF-2α are expressed in the cell cytoplasm of both the glomerular and tubular epithelial cells of patients with classes II, III, or IV than those of patients with class I. HIF-1α and HIF-2α expression was colocalized in the glomeruli of patients with severe glomerular lesions. The HIF-1α and HIF-2α expression were higher in both the glomeruli and tubules of patients in classes II, III, or IV than in those of patients in class I. The incremental value of the addition of HIF-1α and HIF-2α immunostaining score to baseline clinical covariates for the prediction of adverse renal outcomes was then further investigated. This showed that the AIC of a model that included HIF-1α or HIF-2α was similar to that for the model that contained only the clinical covariates (Supplementary Table 5).

## Discussion

The principal findings of the present study are that anemia is an independent risk factor for the progression to ESRD in patients with type 2 diabetes and biopsy-confirmed DN. Each 1 g/dL decrease in baseline Hb was found to be associated with a 42% higher risk of an adverse renal outcome. The renal structural changes were more severe in patients with anemia than in those without anemia. In particular, high RPS DN glomerular classification was closely associated with baseline low Hb. Finally, the addition of the severity of glomerular lesions increased the prognostic value of anemia status for the prediction of the patient’s progression to ESRD.

A retrospective cohort study of 113 patients with DN, but normal Hb or mild anemia conducted in Japan showed that the severity of tubulointerstitial lesions was significantly associated with the progression of anemia in patients with DN [26]. However, patients with moderate or severe anemia, which comprise approximately 50% of those with DN [27], were not included in this study. In the present study, we enrolled patients with differing severity of anemia and showed that the severity of their glomerular lesions was closely associated with low Hb after adjustment for renal function. Herein, the IFTA remained significantly associated with renal outcome in the univariable Cox proportional analysis, but the AIC of adding IFTA to the basic model was slightly higher than that adding RPS classification and/or anemia. This result suggests that renal outcomes correlate more strongly with the presence of changes in glomerular integrity than with deterioration in tubular and interstitial architecture [28,29]. Therefore, anemia, which was associated with glomerular lesions and interstitial changes seems to be a final common pathway to ESRD.

The underlying mechanism linking glomerular lesions to renal anemia is that low Hb causes hypoxia and lower blood flow [30], and the high expression of HIF-1α and HIF-2α in the glomeruli of patients with severe glomerular lesions is consistent with this. Several previous studies have shown that HIF-1a and HIF-2a are upregulated in the kidneys of animals and patients with DN [10,31,32]. Chronic ischemia and hypoxia in the presence of renal interstitial fibrosis have been shown to result in high expression of HIF-1a and HIF-2a, and angiotensin-II increases the expression of HIF-1a in renal tubular epithelial cells [11]. Tubular HIF-2a activation during the late stages of kidney disease protects the kidney against the progression of renal fibrosis [32]. High HIF-1α may promote glomerular scarring, while the knockout of HIF-1α has been shown to be protective against glomerulosclerosis and glomerular type-I collagen accumulation in a mouse model of podocyte-specific HIF-1α ablation [33]. However, hypoxia in the renal interstitium causes impaired production of erythropoietin by reducing the number of peritubular fibroblasts [34]. The reduction or loss of the ability of activated fibroblasts to produce erythropoietin is a major cause of renal anemia in kidney injury, which exacerbates renal fibrosis.

Previous epidemiologic studies have shown that low Hb is closely associated with a decline in renal function in CKD [35]. Low Hb in patients with type 2 diabetes but no clinical albuminuria may be a significant predictor of the subsequent decline in GFR [30]. Recent reports have suggested that anemia is an important risk factor for progression to ESRD in patients with CKD, whether or not they have diabetes [36,37]. However, few studies have investigated the combined effect of renal pathology and Hb on renal outcomes in patients with diabetes. Herein, we have provided evidence that the risk of adverse renal outcomes increases from low in patients with severe glomerular lesions who do not have anemia to higher in those with mild anemia, and further increases in those with moderate or severe anemia, even after adjustment for baseline renal function and pathologic covariates. Furthermore, the adjusted HR in patients with moderate or severe anemia and severe glomerular lesions was the highest in the cohort as a whole. The high risk of progression to ESRD in patients with anemia was even higher in patients with severe glomerular lesions than in those with mild lesions. The present findings suggest that the management of anemia should be stricter in patients with severe glomerular lesions than in those with mild lesions and that this may delay the progression to ESRD in patients with type 2 diabetes.

In a multi-center epidemiologic study conducted in China, anemia was present in 51.5% of patients with CKD who were not undergoing dialysis [2], and it was more prevalent in patients with DN (68.0%) than in patients with hypertensive renal damage (56.6%) or chronic glomerulonephritis (46.1%) [2]. A cross-sectional study conducted in Shanghai, eastern China, showed a prevalence of anemia of 40%–60% in patients with type 2 diabetes and DN [27], whereas in the present study, the prevalence of anemia was as high as 65.0%, and 31.2% even in patients with stage 1 disease. Thus, the prevalence of anemia significantly increased with the increasing CKD stage in patients with diabetes. The present findings suggest a worsening situation in Chinese patients with type 2 diabetes, especially in western China. Therefore, therapy for anemia could be of significant benefit at both the patient and community levels. The risk of anemia in patients with diabetes is estimated to be two to three times higher than that in patients without diabetes [38,39]. Sympathetic denervation of the kidney because of autonomic neuropathy [40], systemic inflammation, and poorer red cell survival compound the anemia in diabetes [41]. Of note, the expression of both HIF-1α and HIF-2α was found to be high in the present study, and these proteins were principally deposited in the cell cytoplasm, rather than in the nucleus, implying that HIF activation is suboptimal in the hypoxic diabetic kidney [42], which would lead to insufficient production of erythropoietin. Roxadustat, a nonselective reversible HIF prolyl hydroxylase inhibitor, ameliorates anemia in patients with CKD and anemia who are not undergoing dialysis [43]. However, because of iron and erythropoietin deficiencies, hyporesponsivenss to erythropoietin, and the hypoxia associated with diabetes [44], the long-term effect of Roxadustat on renal outcomes in patients with diabetes and CKD requires further study.

The 2012 KDIGO guidelines recommend considering the use of ESAs in adult patients with CKD who are not undergoing dialysis and with Hb < 10 g/dL [45, 46]. Of note, only 19.5% of the patients with anemia in the present cohort were being treated using ESA or iron supplementation. Furthermore, the percentage of patients who achieved the target Hb, which was based on the recommendations of the 2007 Kidney Disease Outcomes Quality Initiative (NKF-KDOQI) guidelines, of 11–12 g/dL [47], was only 12.5% (data not shown). However, approximately 22.8% of the patients with CKD stages 1–4 and anemia reported being treated for anemia in a study conducted in the USA [3]. Furthermore, a nationwide observational study conducted in Japan showed that 32.4% of patients with CKD stages 3–5 and anemia were undergoing ESA therapy, with 30.1% of them achieving their targets [48]. These findings suggest that the treatment of anemia in China is relatively inadequate and should be more proactive. Moreover, the shortening of red blood cell lifespan because of substantial impairment in red blood cell deformability, combined with irreversible hypoalbuminemia, renders the treatment of anemia more difficult in patients with diabetes than in those without [49,50]. Abnormal iron metabolism also makes this anemia more difficult to correct in patients with DN [51]. Long-term dietary therapy, in combination with gastrointestinal dysfunction, leads to the abnormal absorption and transport of iron in the small intestine, ultimately resulting in low serum iron concentration and functional iron deficiency in patients with DN. These pathophysiologic changes exacerbate renal anemia and limit its treatment in patients with DN. In summary, the results of the present study emphasize that clinical awareness of the risk of anemia is important for patients with diabetes and associated DN.

The present study had several limitations. First, there was inherent selection bias because only patients who had undergone renal biopsy were studied. Second, the patients who underwent renal biopsy were mainly in glomerular class III, and their IFTA was mainly scored 1 or 2; therefore we could not fully elucidate the relationship between anemia and renal outcomes in patients in the early stages of DN. Third, the baseline Hb was measured on a single occasion at the time of biopsy, which might have led to the misclassification of some of the patients. Forth, because of the retrospective design of the study, serum erythropoietin concentration could not be assessed, and therefore the effect of serum erythropoietin on renal outcomes could not be evaluated.

In summary, in the present retrospective study, we have shown that severe glomerular lesions are closely associated with low Hb. Furthermore, the renal expression of HIF-1α and HIF-2α increases with the severity of the glomerular lesions. We found that low Hb is an independent predictor of adverse renal outcomes. Mild or moderate/severe anemia is significantly associated with a higher risk of ESRD in patients with severe glomerular lesions than in those with mild lesions. Finally, the addition of the RPS glomerular classification improves the value of anemia for the prediction of adverse renal outcomes in patients with type 2 diabetes and associated DN.

## Data Availability

Datasets are available from the corresponding author on reasonable request.
